# Research on emergency logistics information traceability model and resource optimization allocation strategies based on consortium blockchain

**DOI:** 10.1371/journal.pone.0303143

**Published:** 2024-05-20

**Authors:** Chuansheng Wang, Zixian Guo, Fulei Shi, Mingyue Chen, Xinyu Wang, Jia Liu

**Affiliations:** 1 School of Management Engineering, Capital University of Economics and Business, Beijing, China; 2 Armed Police Command Academy, Tianjin, China; Jazan University, SAUDI ARABIA

## Abstract

In response to increasingly complex social emergencies, this study realizes the optimization of logistics information flow and resource allocation by constructing the Emergency logistics information Traceability model (ELITM-CBT) based on alliance blockchain technology. Using the decentralized, data immutable and transparent characteristics of alliance blockchain technology, this research breaks through the limitations of traditional emergency logistics models and improves the accuracy and efficiency of information management. Combined with the hybrid genetic simulated Annealing algorithm (HGASA), the improved model shows significant advantages in emergency logistics scenarios, especially in terms of total transportation time, total cost, and fairness of resource allocation. The simulation results verify the high efficiency of the model in terms of timeliness of emergency response and accuracy of resource allocation, and provide innovative theoretical support and practical scheme for the field of emergency logistics. Future research will explore more efficient consensus mechanisms, and combine big data and artificial intelligence technology to further improve the performance and adaptability of emergency logistics systems.

## Introduction

As globalization accelerates, societal activities are becoming more complex, leading to frequent occurrences of various emergency events. These events underscore the crucial role of emergency logistics in ensuring rapid response and timely resource allocation [1–4]. However, the current emergency logistics system faces significant challenges, such as information asymmetry, a lack of process transparency, and difficulties in assigning responsibilities, all of which severely impact its efficiency and reliability [5–7]. In response to these challenges, consortium blockchain technology, with its distinctive features of decentralization, tamper-proof data, and high transparency, offers a novel solution. Particularly, it demonstrates immense potential in enhancing collaboration among multiple organizations, ensuring the accuracy of data verification, and improving the transparency of the entire process.

This study aims to explore the application of consortium blockchain technology in the traceability of emergency logistics information, focusing on optimizing the data provenance model and consensus mechanism design to better meet the practical needs of emergency logistics. Kumar et al. (2023) highlight how advanced blockchain applications can further enhance process transparency and operational efficiency across complex logistical frameworks, making a significant impact on the management of emergency events [8]. Zhang Y have elaborated on the importance of quickly deploying key resources in emergency events, while also pointing out the problems of infor-mation asymmetry and lack of process transparency [9–12]. Chen B and others have further investigated how these issues lead to resource wastage and delays in response in prac-tical operations. Blockchain technology is considered a potential solution to these problems [13–15]. Liu D and others have conducted in-depth discussions, particularly ana-lyzing how the decentralized and immutable nature of blockchain can enhance data security in the overall supply chain [16–18]. Specifically, consortium blockchain, with its semi-public, multi-party managed model, has been proven by Liao C to be partic-ularly effective for data sharing and verification [19, 20]. However, there are challenges in directly applying it to emergency logistics. Pan H Y has noted that in environments with high data turnover, the consensus mechanism of consortium blockchain needs to be optimized to ensure efficiency and security [21–25].

Building upon existing research in the field of emergency logistics, this study delves into the potential of consortium blockchain technology in enhancing the traceability of emergency logistics information and optimizing the consensus mechanism. Contrasting with prior research, our work focuses on how consortium blockchain technology can optimize the transparency and efficiency of information flow in emergency logistics. Additionally, we have conducted an in-depth study on optimizing the consensus mechanism of consortium blockchain to improve the efficiency and security of the emergency logistics system in high data turnover environments. These aspects have not been thoroughly explored in previous studies, thus making our research innovative and significant both theoretically and practically. Not only does our study propose new solutions, but it also validates the effectiveness of these solutions through simulation experiments, providing valuable references for future improvements in emergency logistics systems.

## Materials and methods

### Requirement quantification and model design

In the contemporary landscape, emergency logistics plays a pivotal role, especially in responding to sudden events [26–29]. From natural disasters to public health emergencies, the rapid and effective distribution and management of resources are crucial for mitigating impacts and safeguarding public safety [30–33]. Despite this, the existing emergency logistics systems frequently encounter challenges such as inefficiencies in resource allocation, resource wastage, and the potential failure of rescue missions, often exacerbated by a lack of transparency and information asymmetry. To overcome these challenges, the integration of Consortium Blockchain Technology proves essential [34–37]. By establishing the Emergency Logistics Information Traceability Model (ELITM-CBT) based on Consortium Blockchain, not only does it enhance the transparency and accuracy of information management but it also significantly improves the operational speed and efficiency of emergency logistics systems. Drawing on the findings from Zhu et al, our model underscores the potential of blockchain to streamline these processes by ensuring data immutability and facilitating real-time updates, critical for effective decision-making during crises [38–40]. Additionally, incorporating methodologies from Korkmaz and Erkayman enhances our model’s capacity to optimize resource allocation and boost the responsiveness of emergency logistics, further contributing to its efficiency [41].

#### Requirements quantification

Emergency logistics, as a unique form of logistics, involves multiple participating entities, various types of goods, and numerous stages of circulation and management. Efficient and accurate emergency logistics are crucial for achieving rapid response and optimal resource allocation in the context of specific emergencies or disaster response processes. However, the transparency, accuracy, and timeliness of emergency logistics information are often affected by various factors. To tackle these challenges, we have developed the Emergency Logistics Information Traceability Model (ELITM-CBT) based on consortium blockchain technology. This model leverages the distributed, decentralized, tamper-proof, and highly transparent characteristics of consortium blockchain to significantly enhance the accuracy and efficiency of information management. By applying consortium blockchain technology in emergency logistics, we not only address the issues of information asymmetry and process opacity but also achieve efficient and fair resource allocation, ensuring the timeliness and effectiveness of emergency logistics responses.

To deeply understand the construction needs of the information tracing model in emergency logistics under consortium blockchain technology, it is first necessary to comprehensively detail the actual operational processes of emergency logistics and its key parameters.

Demand Analysis and Identification:After the occurrence of an emergency, it is necessary to accurately identify the types, quantities, timing, and locations of the required materials. Let the set of required materials be, *D* = {*D*_1_, *D*_2_, …, *D*_*n*_} where each materiali *D*_*i*_ ncludes quantity *Q*_*i*_, type, *T*_*i*_ demand location, *P*_*i*_ and demand time *t*_*i*_.

Resource Pool Establishment:The materials involved in emergency logistics are distributed across multiple storage points. Let the set of storage points be *W* = {*W*_1_, *W*_2_, …, *W*_*m*_} where each storage point *W*_*j*_ includes material types *T*_*j*_, quantities *Q*_*j*_, geographic locations *P*_*j*_, and available allocation times *t*_*j*_.

Logistics Path Planning:Based on the demands and resources, rational logistics paths need to be planned. Let the set of logistics paths be *R* = {*R*_1_, *R*_2_, …, *R*_*k*_}where each path *R*_*l*_ includes starting point *S*_*l*_, endpoint *E*_*l*_, set of waypoints *N*_*l*_ estimated duration *T*_*l*_ cost *C*_*l*_, and risk factors *F*_*l*_.

Information Recording and Verification:During the logistics process, the circulation information of materials needs to be recorded and verified in real-time. Let the set of logistics information records be *L* = {*L*_1_, *L*_2_, …, *L*_*o*_} where each record *L*_*p*_, includes material *ID*_*p*_, circulation start, *S*_*p*_ circulation end, *E*_*p*_ circulation time *t*_*p*_, circulation cost, *C*_*p*_ and circulation status *St*_*p*_.

Data On-Chain and Consensus:Upload the logistics information records to the blockchain and verify them through the consortium blockchain’s consensus mechanism. Let the set of data blocks be, *B* = {*B*_1_, *B*_2_, …, *B*_*o*_} where each block *B*_*r*_ includes a set of information records, *L*_*r*_ generation time *t*_*r*_, hash value of the previous block *H*_*r*−1_, current block hash value *H*_*r*_, and consensus verification time *CT*_*r*_.

Real-Time Monitoring and Dispatch Decision: Based on the on-chain data, monitor the logistics process in real-time and make dispatch decisions. For example, if a blockage is detected in a logistics path, *R*_*l*_ a re-planning of the path can be initiated.

Information Traceability and Emergency Response:During or after the logistics process, information can be traced through the blockchain to respond to sudden changes or for post-event analysis. For example, if there is an issue with a material, *D*_*i*_ its circulation path, *R*_*l*_ storage point, *W*_*j*_ and information record *L*_*p*_ can be traced back.

#### Model assumptions explanation

To construct a viable and precise emergency logistics information traceability model based on consortium blockchain technology, it is necessary to first establish a set of assumptions and constraints. These will lay the foundation for subsequent model construction and algorithm design. The assumptions and constraints are analyzed in detail from the aspects of logistics demand, logistics resources, route planning, information flow, and data uploading to the blockchain.

*Demand stability assumption*. In a short time window (e.g., the initial phase of emergency response), we assume that the demand *D*_*i*_ in terms of quantity *Q*_*i*_, type *T*_*i*_, location *P*_*i*_, and time *t*_*i*_ is relatively stable.

*Demand priority assumption*. Depending on the urgency of the emergency response, different demands *D*_*i*_ have different priorities *Pr*_*i*_.

*Resource schedulability assumption*. It is assumed that the materials at the storage point *W*_*j*_ are available within a feasible allocation time, *t*_*j*_ and resource scheduling can be completed within a certain time frame.

*Resource completeness assumption*. It is assumed that the type *T*_*j*_ and quantity *Q*_*j*_ of materials at each storage point kW *W*_*j*_ nown and accurate.

*Path connectivity assumption*. It is assumed that there is at least one logistics path *R*_*l*_ between any two storage points *W*_*a*_ and *W*_*b*_.

*Path cost constraint*. For each logistics path *R*_*l*_, its cost *C*_*l*_ must meet a predetermined cost ceiling *C*_*max*_.

*On-chain timeliness assumption*. Logistics information records *L*_*p*_ need to be uploaded to the blockchain within a certain time *t*_*up*_ after generation.

*Consensus mechanism assumption*. Nodes in the consortium blockchain follow a uniform consensus mechanism, able to validate and reach consensus on data block *B*_*r*_ within a certain time *CT*_*r*_.

*Monitoring completeness assumption*. Any anomalies or changes in the logistics process can be captured by the real-time monitoring system.

*Decision-making real-time assumption*. In the event of anomalies detected during the logistics process, dispatch decisions can be made and executed swiftly.

#### Model construction

To address the issues mentioned above, we have developed a sophisticated information traceability model for emergency logistics under the framework of consortium blockchain technology. This integration of consortium blockchain technology is pivotal in enhancing the emergency logistics system by ensuring data immutability and streamlining the information verification process. This study delves into blockchain-related factors, especially how the time of recording to the blockchain, consensus time, and data blocks influence the efficiency of the model operation and the accuracy of the results. We meticulously analyze the application of blockchain technology to improve the traceability and reliability of logistics information, showcasing the potential of blockchain in revolutionizing emergency logistics management.

Symbol Explanation:

- *D*_*i*_: The *i*^*th*^ logistics demand point;
- *Q*_*i*_, *T*_*i*_, *P*_*i*_, *t*_*i*_, *Pr*_*i*_: Represent the demand quantity, type, location, time, and priority of the demand point *D*_*i*_ respectively;
- *W*_*j*_: The *j*^*th*^ warehouse point;
- *Q*_*j*_, *T*_*j*_, *t*_*j*_: Represent the inventory quantity, type, and available dispatch time of the warehouse point *W*_*j*_ respectively;
- *R*_*l*_: The *l*^*th*^ logistics route;
- *T*_*l*_, *C*_*l*_: Represent the estimated duration and cost of the logistics route *R*_*l*_, respectively;
- *L*_*p*_: The *p*^*th*^ logistics information record;
- *ID*_*p*_, *S*_*p*_, *E*_*p*_, *t*_*p*_, *C*_*p*_, *St*_*p*_: Represent the item ID, starting point, endpoint, transit time, transit cost, and transit status of the logistics information record *L*_*p*_, respectively;
- *t*_*up*_, *CT*_*r*_, *B*_*r*_: Represent the time of recording to the blockchain, consensus time, and data block, respectively;
- *X*_*ijl*_: A decision variable, indicating whether to send materials from warehouse point *W*_*j*_ to demand point *D*_*i*_ via route *R*_*l*_. *X*_*ijl*_ = 1 if materials are sent, and 0 otherwise.

Establishing the Model. Our objective is to optimize logistics distribution costs, meet demand priorities, reduce transit times, and ensure the accuracy and timeliness of information traceability. To this end, we have constructed a complex multi-objective optimization function(1):

$$\begin{array}{c} Minimize\;Z=\alpha (\sum_{i,j,l}C_{l}x_{ijl})+\beta (\sum_{i,j,l}T_{l}x_{ijl})+\gamma (\sum_{i,j,l}\frac{1-Pr_{i}x_{ijl}}{t_{up}+CT_{r}})+\delta (\sum_{i,j,l}\frac{C_{p}y_{pl}}{\int_{0}^{t_{p}}St_{p}dt}) \end{array}$$ (1)

Subject to:

$$\begin{array}{c} \sum_{j,l}Q_{j}x_{ijl}\geq Q_{i},\forall i \end{array}$$ (2)

$$\begin{array}{c} \sum_{i,l}Q_{i}x_{ijl}\geq Q_{j},\forall j \end{array}$$ (3)

$$\begin{array}{c} \sum_{l}x_{ijl}\geq 1,\forall i,j \end{array}$$ (4)

$$\begin{array}{c} \sum_{l}y_{pl}\geq 1,\forall p \end{array}$$ (5)

$$\begin{array}{c} \sum_{i,j,l}C_{l}x_{ijl}\leq C_{max} \end{array}$$ (6)

$$\begin{array}{c} t_{p}+t_{up}\leq T_{l},\forall p,l \end{array}$$ (7)

$$\begin{array}{c} CT_{r}\leq t_{consensus},\forall r \end{array}$$ (8)

$$\begin{array}{c} {ID}_{p},S_{p},E_{p},t_{p},C_{p},{St}_{p}\neq NULL,\forall p \end{array}$$ (9)

In Eq (1), the weight coefficients *α*, *β*, *γ*, and *δ* are used to balance the importance of various objectives; Eq (2) ensures that the demands of all demand points are met; Eq (3) ensures that the supplies at the storage points are not over-allocated; Eq (4) ensures that at least one path is selected between each demand point and storage point; Eq (5) ensures that the total cost does not exceed the budget; Eq (6) ensures that logistics information is timely recorded on the blockchain; Eq (7) ensures that consensus on the data block is achieved within a certain time frame; Eq (8) ensures the integrity and authenticity of logistics information.

#### Algorithm design

Having constructed an emergency logistics information tracing model under consortium blockchain technology, we recognized the necessity for a uniquely tailored algorithm to address this model’s complex, multi-objective challenges. We innovatively designed and utilized a Hybrid Genetic Algorithm and Simulated Annealing (HGASA), specifically combining the strengths of Genetic Algorithms (GA) and Simulated Annealing (SA) to uniquely address the specific needs of the ELITM-CBT model, showcasing a novel approach in optimizing emergency logistics processes.

Genetic Algorithm is a heuristic search algorithm that mimics the process of natural selection to solve optimization problems. Simulated Annealing, on the other hand, is a probabilistic technique that simulates the cooling process of physical annealing to solve optimization issues. The hybrid algorithm amalgamates the global search capabilities of GA with the local search prowess of SA, aiming to circumvent local optima and enhance solution quality. The algorithmic steps are as follows:

Initialization. Generate an initial population of size *N*. Each individual comprises decision variables *x*_*ijl*_ and *y*_*pl*_ representing logistics distribution and information recording strategies. Additionally, we introduce domain-specific heuristic knowledge to guide the generation of the initial population towards potential optimal solution regions, accelerating convergence speed and increasing the probability of finding optimal solutions;

Fitness Evaluation. For each individual *I*_*k*_, compute the value of the objective function *Z*(*I*_*k*_) leading to a fitness evaluation. This incorporates weight factors for cost, time, priority, and information accuracy, denoted as *α*, *β*, *γ*, *δ*;

Selection. Apply the roulette wheel selection method. Individuals are chosen based on their fitness levels, with higher fitness individuals having a greater probability of being selected;

Crossover. Randomly select pairs of individuals for crossover. Each pair undergoes single or multi-point crossover operations at a given crossover probability *P*_*c*_ producing new offspring.Adaptive methods are employed to dynamically adjust the crossover and mutation probabilities based on the algorithm’s progress, enhancing the population’s exploration and exploitation capabilities;

Mutation. Mutate gene positions of individuals at a certain mutation probability *P*_*m*_ to enhance population diversity;

Simulated Annealing Adjustment.Conduct local search on selected individuals. For each *I*_*k*_, perform the following:

Randomly tweak a decision variable to generate a new individual $I_{k}^{\prime }$;

Compute the objective function value $Z(I_{k}^{\prime })$;

Determine acceptance of the new individual using simulated annealing criteria: if $Z\left(I_{k}^{\prime }\right)<Z\left(I_{k}\right)$, accept the new individual, replacing *I*_*k*_ with $I_{k}^{\prime }$; if $Z\left(I_{k}^{\prime }\right)\geq Z\left(I_{k}\right)$, accept the new individual with a certain probability that varies with the temperature parameter *T* and the difference $Z\left(I_{k}^{\prime }\right)-Z\left(I_{k}\right)$;

Update the temperature parameter to *T* × cooling rate where the cooling rate is typically less than 1.

Replacement. Implement an elitist strategy, retaining a certain proportion of the fittest individuals in the current population, while replacing the rest with newly generated individuals.This elite retention mechanism ensures that optimal solutions are not lost over generations.

Termination Condition Check. Verify if termination criteria are met: if the maximum number of generations is reached or the change in fitness is below a preset threshold, the algorithm stops. Repeat steps 2 to 8 until these conditions are satisfied.

The HGASA algorithm capitalizes on GA’s global search capability and SA’s local search ability. Through intricately designed crossover, mutation, and simulated annealing adjustments, it addresses the multi-objective optimization problems of emergency logistics information tracing under consortium blockchain technology. The innovation lies in enhancing GA results with SA optimization, improving solution quality and avoiding local optima.

In terms of complexity analysis, the algorithm’s time complexity depends on population size *N*, number of generations *G*, length of individual genes *L*, and the number of annealing steps *S* in SA. Thus, the overall time complexity is approximately *O*(*N* ⋅ *G* ⋅ *L* ⋅ *S*) while the space complexity mainly depends on population storage, estimated as *O*(*N* ⋅ *L*).

### Optimization of consensus mechanisms

Applying consortium blockchain technology in emergency logistics information tracing presents several challenges, with the selection of an appropriate consensus algorithm being the most formidable. Through a comparative analysis of mainstream consensus mechanism features (Table 1), it becomes evident that Proof of Work (PoW) and Proof of Stake (PoS) algorithms, which are suitable for public blockchains with a large number of nodes, are not ideal for the Emergency Logistics Information Tracing Model using Consortium Blockchain Technology (ELITM-CBT). This is due to their significant computational power requirements, complex network configurations, and token mechanisms.

**Table 1:** Comparative analysis of mainstream consensus mechanism features.

	POW	POS	PBFT	POA	PAXOS
Decentralization	Strong	Strong	Medium	Medium	Weak
Identity Verification	No	No	Yes	Yes	Yes
Block Node Dynamics	Possible	Possible	By Node Identity	By Node Identity	By Node Identity
Byzantine Fault Tolerance	50%	50%	33%	33%	33%
Suitable Chain	Public	Public	Consortium	Consortium/Private	Private
Resource Consumption	High	High	Low	Low	Low
Consistency	Yes	Yes	Yes	Cannot Guarantee	Yes

Detailed descriptions and notes regarding the comparative analysis of consensus mechanism features can be added here.

The Practical Byzantine Fault Tolerance (PBFT) and Proof of Authority (PoA) algorithms, commonly utilized in consortium blockchains, face constraints in terms of the number of participating nodes and data consistency, rendering them unsuitable for the Emergency Logistics Information Traceability Model (ELITM-CBT) based on consortium blockchain. To overcome these challenges, and acknowledging the frequent changes in emergency supply entities as primary nodes, we have developed a novel consensus algorithm specifically tailored for ELITM-CBT, named C-PBFT. This algorithm is an enhancement of the PBFT algorithm, integrated with the primary node rotation concept from the Clique algorithm, optimizing adaptability to dynamic network conditions and strengthening the assurance of data consistency.

#### Selection of the primary node

In the C-PBFT consensus mechanism, the selection of the primary node is updated at the block height of each target block. Additionally, C-PBFT incorporates a view mechanism when calculating the primary node to address Byzantine problems that may arise with the primary node. The formula for calculating the primary node is as follows (10). In the formula, *P*_*i*_ represents the primary block node; *h* represents the block height; *v* represents the view number; |*R*| represents the number of authoritative nodes.

$$\begin{array}{c} P_{i}=\left(h+v\right)\;\text{mod}\;\left|R\right| \end{array}$$ (10)

#### Block verification

**Fig 1:**
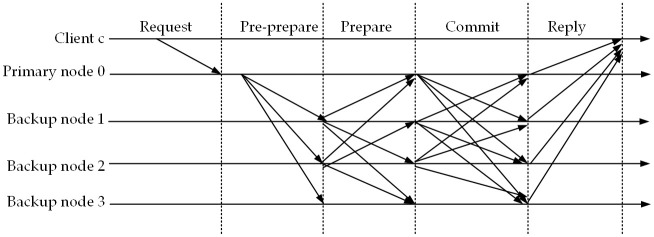
Block verification and commit process. The figure illustrates the process of block verification in the C-PBFT consensus mechanism. A: Client Request phase. B: Pre-Preparation phase. C: Preparation phase. D: Commit Preparation phase. E: Commit and Chain phase.

In the C-PBFT consensus mechanism, the ELITM-CBT clients send a request to the primary node block, which is then followed by the proposal of the block by the primary node currently in rotation. The backup nodes will go through two rounds of voting to validate and confirm the block before it is finally committed to the chain(Fig 1).

1. Client Request: The client *c* sends a request to the primary node 0;
2. Pre-Preparation: Upon receiving the request, the primary node 0 broadcasts a pre-prepare message to the backup nodes at the current block height *h*;
3. Preparation: The backup nodes, after receiving the pre-prepare message, save and validate it within the allowed time frame, and then broadcast a prepare message;
4. Commit Preparation: During the prepare phase, if a node receives 2*f* + 1 matching prepare messages, it broadcasts a commit message to the other nodes and enters the commit phase;
5. Commit and Chain: In the final commit phase, if a node receives 2*f* + 1 commit messages from other nodes, it proceeds with the block commitment to the chain.

The C-PBFT consensus mechanism requires that the total number of honest nodes in the network satisfies *N* ≥ 3*f* + 1, which means $f\leq \frac{N-1}{3}$. This implies that the model can tolerate up to $\frac{N-1}{3}$ faulty nodes. Clearly, when the primary node is honest and there are at most $f\leq \frac{N-1}{3}$ faulty nodes, the C-PBFT consensus mechanism can continue to operate normally. This also ensures the fault tolerance and stability of the ELITM-CBT system.

#### View change mechanism

**Fig 2:**
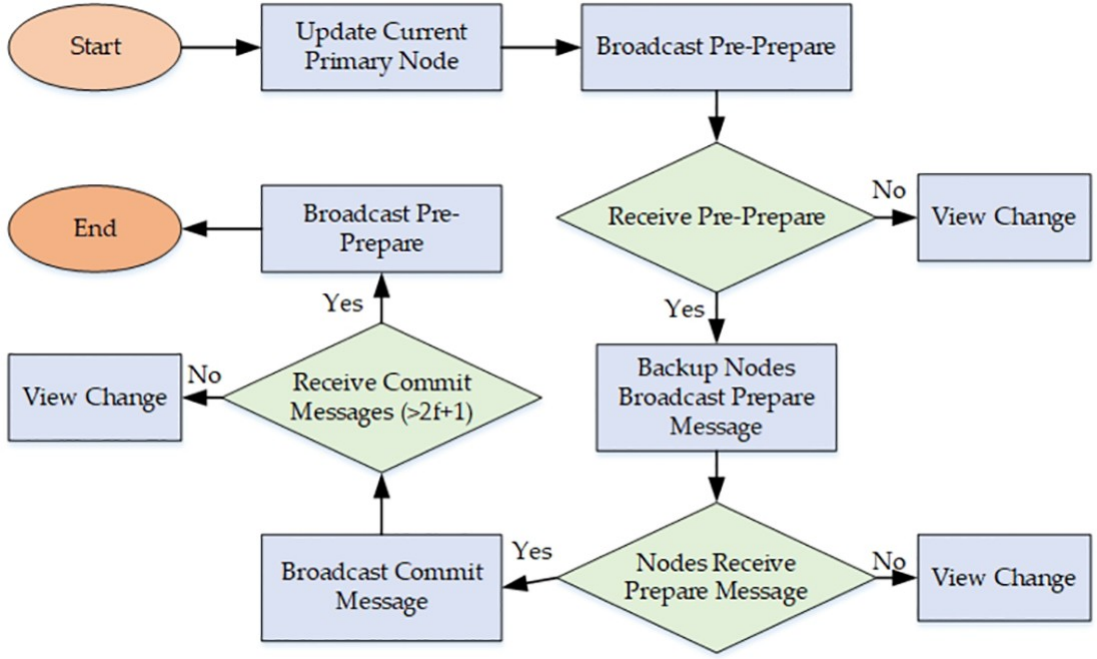
C-PBFT consensus mechanism and view change flowchart.

When the primary node encounters an error, a view change mechanism is triggered. With each update of the block height, all nodes start a timer. If a backup node does not receive a message from the primary node, or if the message from the primary node is erroneous and consensus cannot be reached within the allotted time, the backup node will broadcast a view-change message. Subsequently, the nodes broadcast a prepare message in the new view. If nodes collect prepare messages from different views and find that it is not possible for 2*f* + 1 nodes to enter the prepare phase, they will not execute the request message. At this point, the calculation for a new primary node is also initiated. The flowchart for triggering the view change mechanism in the C-PBFT consensus mechanism when the primary node is faulty is illustrated in Fig 2.

#### C-PBFT consensus process

The C-PBFT consensus process is divided into three main phases: pre-prepare, prepare, and commit. There are two main conditions for rotating the primary node: one is the normal cyclical rotation, and the other is the update of the primary node using the view change mechanism due to non-response or erroneous behavior by the primary node. The main steps of the C-PBFT consensus process are as follows:

1. Client Request: Client (c) sends a message to the nodes with the structure 〈request, *o*, *t*, *c*〉. Here, ‘request’ stands for the content, ‘o’ for the operation requested, ‘t’ for the timestamp, and ‘c’ for the client’s identifier, along with the client’s signature.
2. Primary Node Broadcast: Nodes calculate whether they are the primary node for the current target block height. If so, they verify the client’s signature. If the signature is incorrect, the request is discarded. If correct, the primary node broadcasts 〈pre- prepare, *h*, *v*, *d*, *r*〉 to the backup nodes, where ‘h’ is the block height, ‘v’ is the view number, ‘d’ is the digest of the request ‘r’, with the primary node’s signature.
3. Backup Node Validation: If a node is not the primary, it checks for a 〈pre- prepare, *h*, *v*, *d*, *r*〉 message from the primary. Upon receipt, it validates the message (verifying the primary’s signature, consistency of ‘d’ with the request digest, and ‘v’ across messages). If validation fails, the message is discarded. If it passes, the backup node saves and broadcasts 〈prepare, *h*, *v*, *d*, *i*〉, with ‘i’ indicating the backup node’s identifier and includes the backup node’s signature, awaiting prepare votes.
4. Commit Broadcast: After receiving *n* ≥ 2*f* + 1 prepare votes, the node broadcasts 〈commit, *h*, *v*, *d*, *i*〉 to other nodes, waiting for their 〈commit, *h*, *v*, *d*, *i*〉 messages.
5. Block Commit: Upon receiving *n* ≥ 2*f* + 1 commit votes, the node proceeds with the block commitment operation.
6. Consensus Interruption and View Change: During the consensus process, if a backup node is non-responsive within a specified time or consensus is not achieved, it broadcasts 〈view change, *h*, *v*, *i*〉 to other nodes, waiting for votes. If the primary node is faulty, the same view change mechanism described above is initiated. When *n* ≥ 2*f* + 1 view change votes are received, the primary node and the corresponding view number are updated.

## Results and discussion

### Simulation experiment

In response to an earthquake disaster in a particular region, this study constructs an emergency logistics information tracing model involving multiple locations. The model consists of several supply and demand points, with logistics transportation between points recorded and monitored using consortium blockchain technology. This ensures the effective allocation of materials and transparency and traceability of information. The model assumes the involvement of the following entities:

- **Supply Points (S)**: Warehouses or logistics centers that provide disaster relief materials.
- **Demand Points (D)**: Disaster areas or relief stations that require disaster relief materials.
- **Materials (M)**: Various types of disaster relief materials, such as medical supplies, food, water, tents, etc.
- **Transport Network**: Transportation routes connecting supply points to demand points.

Each supply point has a certain reserve of materials, while each demand point has a certain demand for materials. Materials are allocated with different priorities based on their urgency. Logistics activities need to be completed within a specific time frame to ensure a rapid response.

#### Case study solution

In this case study, we employed multi-objective optimization methods to solve the emergency logistics distribution problem. Our goals include minimizing total transportation time, minimizing total cost, and maximizing the fairness of material distribution. Considering the complexity of actual emergency logistics scenarios, we introduced an improved consensus algorithm to ensure the rapid and accurate transmission of information.

Simulation Environment. The simulation experiment was conducted on a computer equipped with an Intel Core i7-10750H CPU @ 2.60GHz and 16GB RAM. All algorithms were written in Py-thon 3.8, using the NumPy library for data operations and calculations, the Pandas li-brary for data management, and graphical displays generated through the Matplotlib and Seaborn libraries.

The simulation experiment is divided into baseline and improved experiments, corresponding to the consensus algorithm before and after improvement. Each part of the experiment was run dozens of times to ensure the stability and accuracy of the sta-tistical results. Statistical analysis of the experiment results was performed using Py-thon’s SciPy library.

All experimental results are recorded in tabular form and presented graphically for easy comparison and analysis. The storage and management of experimental re-sults utilized the Git version control system, ensuring the integrity and traceability of the experimental data.

In this study, we have conducted a detailed analysis of the ELITM-CBT model’s effectiveness and its performance under two different consensus mechanisms: PBFT (the baseline experimental scheme (Table 2)) and C-PBFT (the improved experimental scheme(Table 3)). Each experimental scheme generated data for ten allocation plans, with performance metrics categorized into total transportation time, total cost, fairness index, consensus time, and throughput.

**Table 2:** Baseline experiment (Before improvement) results.

Plan ID	Total Time (hours)	Total Cost	Fairness Index	Consensus Time	Throughput
1	76.99	7762.91	0.87	0.09	159.73
2	77.38	7786.20	0.75	0.06	240.00
3	75.43	7952.31	0.65	0.11	136.64
4	76.69	7075.43	0.77	0.06	239.98
5	73.58	8171.77	0.84	0.08	191.04
6	64.22	7254.01	0.80	0.08	192.01
7	73.87	8728.99	0.74	0.08	191.94
8	70.62	8573.21	0.84	0.06	239.97
9	63.21	7638.34	0.85	0.06	239.99
10	72.53	7332.19	0.82	0.08	190.75

Baseline experiment results description here.

**Table 3:** Improved experiment (After improvement) results.

Plan ID	Total Time (hours)	Total Cost	Fairness Index	Consensus Time	Throughput
1	75.43	8059.99	0.77	0.11	137.17
2	70.64	7821.81	0.85	0.03	461.63
3	73.25	7986.11	0.83	0.06	238.97
4	61.63	6814.96	0.76	0.06	234.37
5	63.04	7388.72	0.75	0.06	233.34
6	77.64	7918.58	0.88	0.06	249.91
7	71.12	7213.98	0.85	0.05	288.49
8	71.92	7353.93	0.92	0.10	152.91
9	78.15	7819.52	0.80	0.11	137.43
10	62.17	7326.70	0.82	0.07	200.08

Improved experiment results description here.

Performance Analysis of C-PBFT and PBFT Consensus Mechanisms. In this research, we delve into a comprehensive analysis of the performance differences between C-PBFT and PBFT consensus mechanisms within the Emergency Logistics Information Traceability Model (ELITM-CBT). The primary objective of this analysis is to evaluate and compare the performance of these two consensus mechanisms in terms of consensus time and throughput. To facilitate an effective comparison, we first define two key performance indicators: consensus time and throughput.

Consensus time refers to the duration required to achieve consensus in the blockchain, serving as a crucial metric for evaluating the efficiency of a consensus mechanism. A shorter consensus time indicates higher efficiency of the consensus mechanism, which, in the context of emergency logistics, means faster processing and confirmation of transactions, thereby accelerating the overall response speed.

Throughput represents the number of transactions that the system can handle per unit of time. A higher throughput denotes the system’s enhanced capability to effectively process a large volume of transactions, which is vital for addressing the extensive needs and complex resource allocation during sudden events.

**Fig 3:**
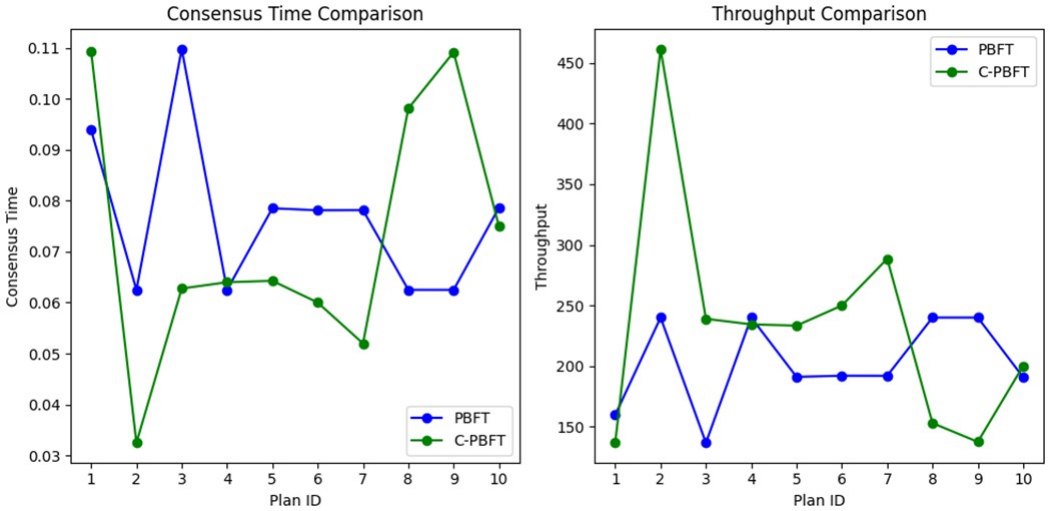
Comparative analysis of consensus time and throughput for PBFT and C-PBFT mechanisms.

Through comprehensive data analysis and visual comparison as illustrated in Fig 3, we highlight the significant optimization role of the C-PBFT consensus mechanism within the emergency logistics information system. The mechanism’s superior performance in consensus time over the PBFT not only reflects higher efficiency but is particularly crucial in the high-stress environment of emergency logistics. Specifically, C-PBFT achieves a notable reduction in decision-making delays by optimizing the consensus process, ensuring that critical supplies and information can be rapidly and accurately distributed and traced in the event of an emergency. Moreover, the enhanced processing capability of C-PBFT—as evidenced by the significant increase in throughput shown in Fig 3—further demonstrates its advantages in scenarios involving high loads and massive transaction processing. These improvements not only strengthen the response capability and processing efficiency of the emergency logistics information system but also, through the application of blockchain technology, enhance the system’s transparency and reliability, providing a more secure, efficient, and transparent solution for emergency logistics management.

Fairness Index Analysis. In our study, the Fairness Index (FI) is utilized as an essential metric to evaluate the efficiency of resource allocation within emergency logistics. The fundamental premise of the Fairness Index is to gauge the equitability of resource allocation across various demand points. Within the sphere of emergency logistics, the equitable distribution of resources plays a pivotal role in ensuring both effective and timely relief operations. The computation of the Fairness Index is predicated on the ratio of the amount of resources allocated to each demand point in relation to its respective requirements, as delineated in Eq (11). Herein, *N* signifies the aggregate number of demand points, *Q*_*a*,*i*_ denotes the quantity of resources allocated to the *i*^*th*^ demand point, and *Q*_*r*,*i*_ is indicative of the requisite amount for the *i*^*th*^ demand point.

$$\begin{array}{c} FI=\frac{1}{N}\sum_{i=1}^{N}\text{min}(\frac{Q_{a,i}}{Q_{r,i}},1) \end{array}$$ (11)

**Fig 4:**
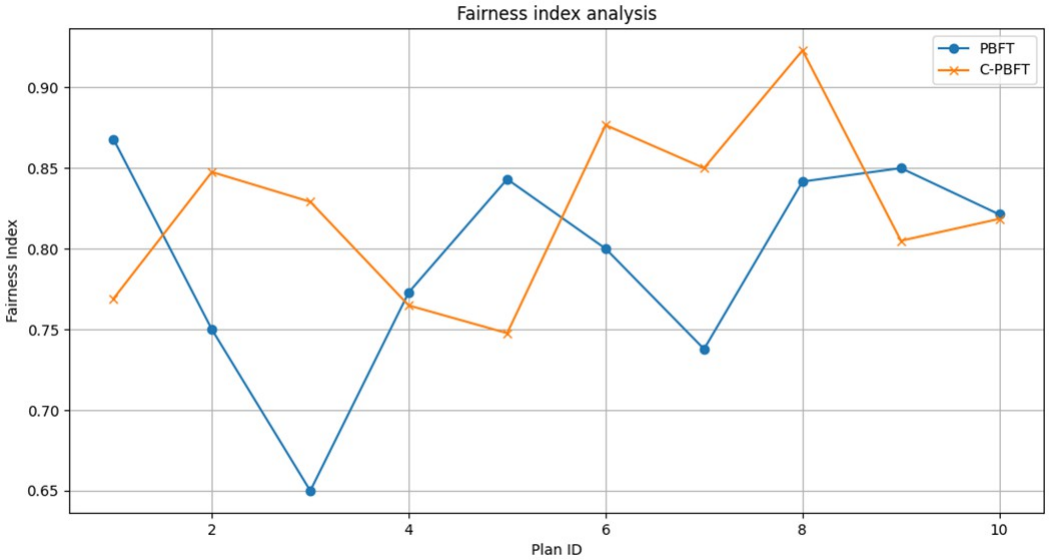
Fairness index analysis.

In order to conduct a visual analysis of the impact exerted by disparate consensus mechanisms on the Fairness Index, line graphs (Fig 4) are employed to demonstrate the fluctuation of the Fairness Index relative to the two data sets under scrutiny. The horizontal axis of the graph is representative of varying plan IDs, whilst the vertical axis depicts the corresponding values of the Fairness Index. Observations from the line graph elucidate that, notwithstanding the presence of individual discrepancies in the Fairness Index under the two consensus mechanisms, the overall levels predominantly exhibit a high threshold, consistently maintaining between 0.8 and 0.9. Such findings attest to the considerable strengths of the ELITM-CBT model in facilitating an equitable resource distribution, thereby ensuring a balanced allocation of resources in emergency scenarios and effectively catering to the distinct requirements of various demand points.

Optimization of Total Transportation Time. To thoroughly assess the optimization of total transportation time within the emergency logistics system, we introduce an improved index that considers weighting factors. This index synthesizes transportation time data from both baseline and improved plans, with weights reflecting the importance of transportation time in different scenarios. This allows for a more comprehensive understanding of how the improved consensus mechanism impacts total transportation time.

$$\begin{array}{c} \Delta T_{\text{opt}}=\sqrt{\frac{\sum_{i=1}^{n}(T_{{\text{base}}_{i}}-T_{{\text{improved}}_{i}}{)}^{2}\times W_{i}}{n}} \end{array}$$ (12)

In this analysis, $T_{{\text{base}}_{i}}$ and $T_{{\text{improved}}_{i}}$ represent the total transportation time for the *i*^*th*^ plan under baseline and improved conditions, respectively, while *W*_*i*_ denotes the importance weight of the *i*^*th*^ plan, and *n* is the total number of plans. This formula (12) measures the degree of optimization in total transportation time by considering the importance of each plan and calculating the weighted squared difference between baseline and improved scenarios.

**Fig 5:**
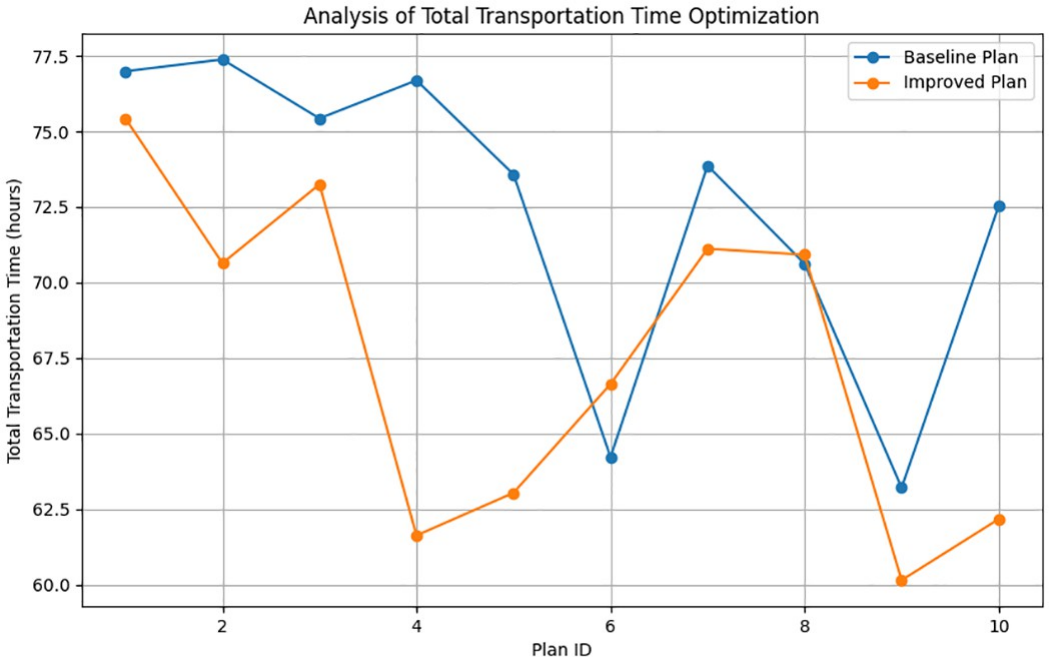
Comparison of total transportation time optimization.

The line graph presented in Fig 5 vividly illustrates the comparison of total transportation time between the baseline and improved schemes. The degree of optimization in total transportation time for each plan is quantified by calculating the weighted squared difference between the two states. This method allows for a more comprehensive revelation of the impact of the improvement strategies on reducing transportation time. Notably, the overall transportation time optimization of the improved plans is consistently shorter than that of the baseline plans. This analysis underscores the effectiveness of the ELITM-CBT model in optimizing the transportation time of emergency logistics systems, particularly when employing the C-PBFT consensus mechanism. Such optimizations contribute significantly to enhancing the efficiency of emergency responses, ensuring that resources are allocated to the required locations in the shortest possible time.

Cost-Benefit Analysis. Cost-benefit analysis is a key aspect of evaluating the effectiveness of improvements in an emergency logistics system. This study introduces an improved index that comprehensively considers both costs and benefits. By examining the cost variability index of each plan, the study more thoroughly assesses the impact of the improved consensus mechanism on cost-effectiveness.

$$\begin{array}{c} \Delta C_{\text{eff}}=\frac{\sum_{i=1}^{n}(\frac{C_{{\text{base}}_{i}}-C_{{\text{improved}}_{i}}}{C_{{\text{base}}_{i}}})\times V_{i}}{n} \end{array}$$ (13)

In this assessment, $C_{{\text{base}}_{i}}$ and $C_{{\text{improved}}_{i}}$ represent the total cost of the *i*^*th*^ plan under baseline and improved conditions, respectively, while *V*_*i*_ is the cost volatility index for the *i*^*th*^ plan, and *n* is the total number of plans. The formula (13) measures the cost-benefit of each plan by calculating the percentage of cost savings and incorporating the cost volatility index.

This method not only considers the absolute amount of cost savings but also looks at the proportion of cost savings relative to the original cost, as well as the cost volatility of each plan. This approach provides a more comprehensive and in-depth cost-benefit analysis, offering a nuanced view of the economic efficiency of the plans.

**Fig 6:**
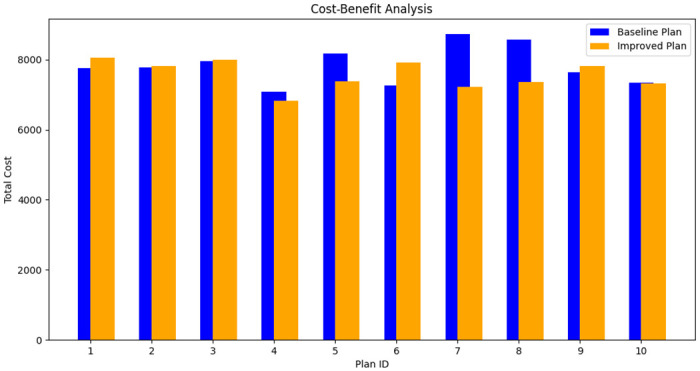
Cost-benefit analysis.

The bar chart (Fig 6) clearly demonstrates the comparison of total costs be-tween the baseline and improved plans. In each group of bars, blue represents the cost of the baseline plan, while orange represents the cost of the improved plan. By com-paring the two sets of bars, it is evident that, in most cases, the improved plan incurs lower costs than the baseline plan. Additionally, considering the cost volatility index, this chart also reflects the resilience of each plan against cost fluctuations. Taller bars indicate that the improved plans can achieve greater cost savings in a fluctuating market environment, thereby enhancing overall cost-effectiveness.

## Conclusion

In this research, we successfully constructed an Emergency Logistics Information Traceability Model (ELITM-CBT) based on consortium blockchain technology, addressing the urgent need for efficient information flow and resource allocation in emergency scenarios. This study has proven the transformative impact of consortium blockchain technology on the emergency logistics field, particularly highlighting its capabilities in ensuring data immutability, enhancing transparency, and achieving accurate traceability. By leveraging the unique features of blockchain, such as decentralization, security, and consensus mechanisms, we provided a comprehensive solution that overcomes the limitations of traditional emergency logistics models.

Our in-depth simulation experiments showcased the ELITM-CBT model’s remarkable improvements in total transportation time, cost efficiency, and equitable resource allocation, underscoring the model’s superior handling of emergency logistics complexities. These findings not only demonstrate the practical applicability and effectiveness of consortium blockchain technology in reshaping emergency logistics but also emphasize its role in advancing the transparency and efficiency of logistical operations. The integration of the Hybrid Genetic Simulated Annealing Algorithm (HGASA) further accentuated the model’s optimization capabilities, showcasing a synergistic blend of algorithmic precision and blockchain’s robustness.

As we look to the future, the integration of consortium blockchain technology within emergency logistics holds tremendous promise. Continuous technological progression promises even more sophisticated consensus mechanisms and blockchain functionalities, aiming to slash operational costs and boost data throughput. Emphasizing the integration of cutting-edge technologies such as big data and artificial intelligence with blockchain will likely herald a new era of innovation in emergency logistics, offering enhanced analytical capabilities and decision-making precision. Such advancements will undoubtedly open new avenues for research, driving forward the development of more resilient, transparent, and efficient emergency logistics systems.
